# Unveiling Competitive Adsorption in TiO_2_ Photocatalysis through Machine-Learning-Accelerated Molecular Dynamics, DFT, and Experimental Methods

**DOI:** 10.1021/acsami.4c02334

**Published:** 2024-07-02

**Authors:** Omar Allam, Mostafa Maghsoodi, Seung Soon Jang, Samuel D. Snow

**Affiliations:** † Woodruff School of Mechanical Engineering, Georgia Institute of Technology, Atlanta, Georgia 30332, United States; ‡ Computational NanoBio Technology Laboratory, School of Materials Science and Engineering, Georgia Institute of Technology, Atlanta, Georgia 30332, United States; § Department of Civil and Environmental Engineering, Louisiana State University, 3255 Patrick Taylor Hall, Baton Rouge, Louisiana 70803, United States

**Keywords:** TiO_2_, heterogeneous photocatalysis, adsorption, density functional theory, competitive adsorption, machine learning interatomic potentials

## Abstract

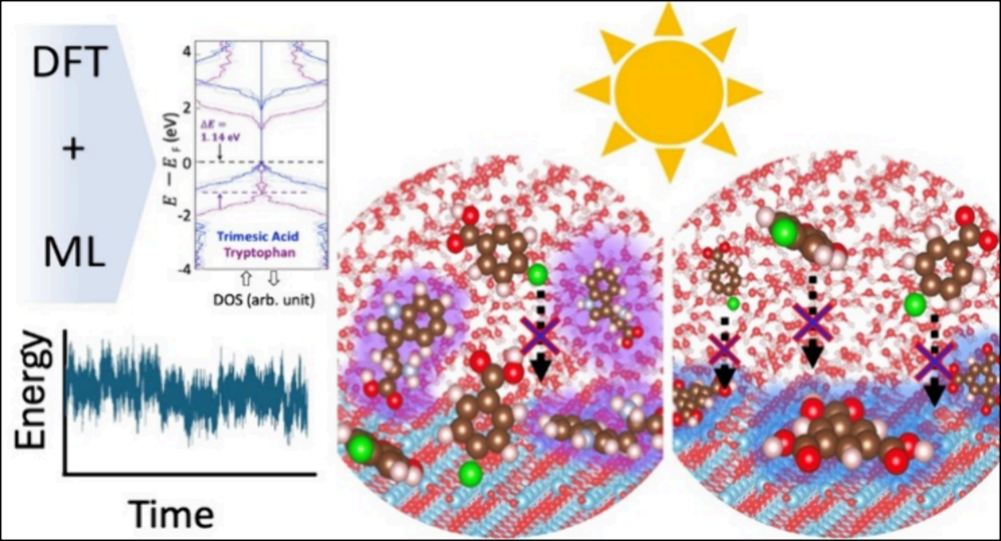

The efficient harnessing of solar power for water treatment via photocatalytic processes has long been constrained by the challenge of understanding and optimizing the interactions at the photocatalyst surface, particularly in the presence of nontarget cosolutes. The adsorption of these cosolutes, such as natural organic matter, onto photocatalysts can inhibit the degradation of pollutants, drastically decreasing the photocatalytic efficiency. In the present work, computational methods are employed to predict the inhibitory action of a suite of small organic molecules during TiO_2_ photocatalytic degradation of *para*-chlorobenzoic acid (*p*CBA). Specifically, tryptophan, coniferyl alcohol, succinic acid, gallic acid, and trimesic acid were selected as interfering agents against *p*CBA to observe the resulting competitive reaction kinetics via bulk and surface phase reactions according to Langmuir–Hinshelwood adsorption dynamics. Experiments revealed that trimesic and gallic acids were most competitive with *p*CBA, followed by succinic acid. Density functional theory (DFT) and machine learning interatomic potentials (MLIPs) were used to investigate the molecular basis of these interactions. The computational findings showed that while the type of functional group did not directly predict adsorption affinity, the spatial arrangement and electronic interactions of these groups significantly influenced adsorption dynamics and corresponding inhibitory behavior. Notably, MLIPs, derived by fine-tuning models pretrained on a vastly larger dataset, enabled the exploration of adsorption behaviors over substantially longer periods than typically possible with conventional ab initio molecular dynamics, enhancing the depth of understanding of the dynamic interaction processes. Our study thus provides a pivotal foundation for advancing photocatalytic technology in environmental applications by demonstrating the critical role of molecular-level interactions in shaping photocatalytic outcomes.

## Introduction

Grand promises of affordable and sustainable water treatment via photocatalytic technologies have, thus far, disappointed.^1^ Despite a seemingly endless stream of advances in visible light absorption, electron–hole separation efficiency, quantum yield, or other material properties, applications of photocatalysis for water treatment are niche at best.^2^ This failure of technology transfer stems from the difficulty in process design for photocatalytic systems. Photocatalyst loading with high surface area must be achieved while maintaining excellent light management for photon delivery, and such a system must be robust against nontarget solutes in complex waters, such as dissolved organic matter (DOM). The ability to produce highly reactive oxygen species (ROS) upon irradiation is perhaps the most compelling and challenging feature of photocatalytic materials. Complex media, however, pose a significant challenge, inhibiting the action of ROS by competitive quenching reactions.^3−7^ The development of strategies to mitigate or avoid inhibition by DOM is therefore critical for successful photocatalytic water treatment.

Comparisons of photocatalytic activity from one material to another have been notoriously difficult over the years. Differences in synthesis, characterization, photochemical reactor designs, and activity assays, among other variables, contribute to this difficulty to this day.^8,9^ The common practice of using probe molecules with known reactivities with ROS to quantify effective steady-state ROS concentrations highlighted a key concept: surface-phase reactions are extremely important. In 2008, Ryu and Choi prescribed a multiactivity assay approach to adequately assess and compare photocatalytic materials.^8^ Differences in ROS-probe molecule rate constants were insufficient to explain differences in photoactivity, as measured by different probes. In 2015, Brame et al. derived and experimentally validated a combined model of surface- and bulk-phase reactions between ROS and target molecules using a modified Langmuir–Hinshelwood isotherm to describe adsorption onto photocatalyst surfaces.^4^ They further demonstrated that competitive quenching dynamics can be described by observing the trend of observed probe-ROS reaction rate constants as a function of the concentration of inhibitory compounds. The nature of that trend correlated with the extent to which the inhibitor competed with the probe on the surface of the photocatalyst. We have since used this approach to characterize the potential for surface fouling by wastewater DOM, finding that membrane bioreactor operation could be optimized to minimize the inhibition of TiO_2_ photocatalysts.^6^ Furthermore, we showed that molecule-specific interactions (as opposed to colloidal stability) dictate the competitive adsorption–inhibition process.^7^

Exploring the inhibition dynamics of photocatalytic systems using this experimental rate-profile approach provides an important complement to multiactivity assays. Both methods, however, require significant experimentation. Computational simulations of molecule–photocatalyst interaction dynamics may provide a predictive tool capable of predicting specific catalytic activity. Density functional theory (DFT) computations have been employed to examine interactions between TiO_2_ and a variety of molecules,^10^ including formaldehyde,^11^ cyclohexanone,^12^ chlorophenols,^13^ and formic acid,^14^ to name a few. Similar work used DFT analyses to test how modifications to semiconductors, either with surface functionalization or doping/vacancy manipulations, affected their adsorptivity or activity.^15^ Thus, DFT may allow for rapid screening of photocatalysts for performance in the presence of inhibitory compounds.

Few studies have employed DFT to predict or understand photocatalytic performance, and none, to our knowledge, have endeavored to describe competitive sorption for aqueous phase reactions. The present study combines a thorough experimental inhibition assay with DFT and machine learning (ML)-accelerated explicit solvation simulations to test the ability of calculated interaction energies to predict photocatalytic inhibition. Experiments tested the TiO_2_-mediated photocatalytic degradation of probe compound *para*-chlorobenzoic acid (*p*CBA) against five different inhibitory molecules, and simulations calculated binding energies for each with a TiO_2_ surface. We thus provide experimental validation of a computational approach to predicting photocatalytic inhibition and delineate moiety-specific adsorption dynamics.

## Results/Discussions

### Competition for OH· in Bulk Solution

**Figure 1:**
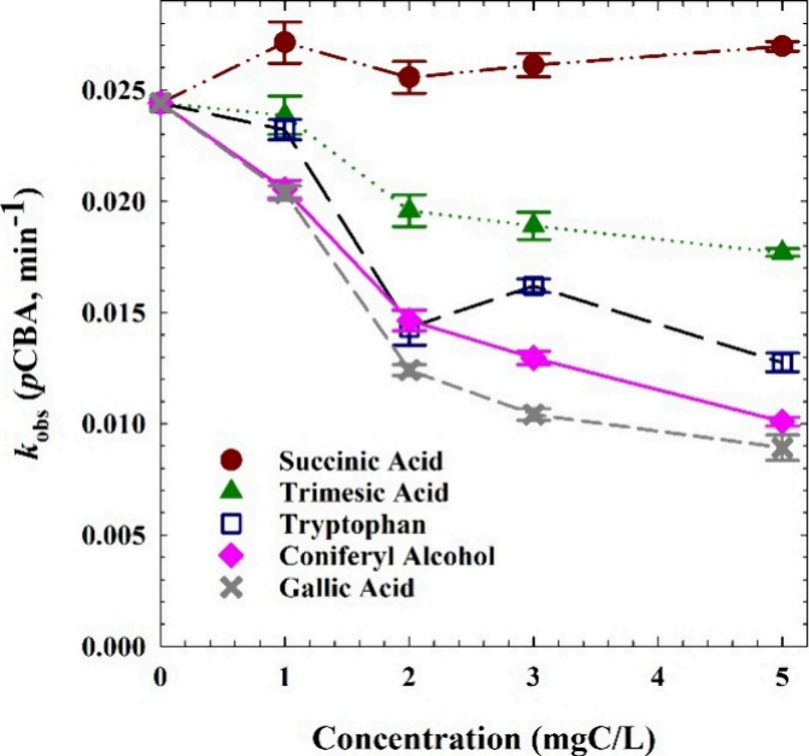
Rate constants for *p*CBA degradation by OH· produced via photolysis of 1 mM H_2_O_2_ under 278 nm irradiation in the presence of varying concentrations of inhibitory molecules.

Hydroxyl radical quenching experiments were performed with *p*CBA and each of the selected inhibitory molecules to delineate their competitive kinetics for reactions with OH· in a bulk solution. Molecular diagrams of these molecules are shown in Figure S1 of the Supporting Information. Destruction of *p*CBA was assumed to be pseudo-first order with respect to an effective steady state [OH·] generated via photolysis of 1 mM H_2_O_2_ solution over 10 min (Figure S2 of the Supporting Information). The resulting observed rate constants (*k*_obs_) for each condition are plotted in Figure 1 as a function of inhibitory molecule concentration. Bimolecular reaction rate constants between OH· and *p*CBA and the quenching agents, except for trimesic acid, have been documented previously.^16−18^ These values are reported in Table 1, and a value for trimesic acid reaction with OH· was estimated based on the observed competitive quenching. To make this estimation possible, the hydroxyl radical concentration was assumed to reach a steady state in the photolytic system, where the rate of generation equals the rates of removal by quenching reactions, with the solvent, with *p*CBA, or with trimesic acid. Likewise, the concentration of trimesic acid was assumed to be constant during the experimental time. In the absence of quenchers, *p*CBA was removed at a pace of 0.0244 min^–1^, which corresponds to an estimated [OH·]_ss_ of 8.1 × 10^–14^ M. Thus, by observing changes in *p*CBA concentration over time in the presence of trimesic acid, a bimolecular rate constant of 4.3 × 10^8^ M^–1^ s^–1^ was derived for the reaction between OH· and trimesic acid. A comparison of the known rate constants and the observed competition profile (Figure 1) shows reasonable agreement with the expected order of inhibition. The competition at 5 mgC/L followed this order: gallic acid > coniferyl alcohol > trimesic acid > succinic acid; the strongest inhibitors performed similarly with bimolecular rate constants near 10^10^ M^–1^ s^–1^. Notably, succinic acid appeared to slightly improve *p*CBA removal, perhaps providing a catalyzing effect. These bulk-phase reactions provide a baseline for comparison to the TiO_2_ system, where bulk and surface-phase reactions are expected.

**Table 1:** Molecular Characteristics and OH· Reactivity of Probe and Inhibitor Compounds

molecule	characteristics	rate constant (M^–1^ s^–1^)	ref
*p*CBA	carboxyl, chloro substituted, aromatic	5 × 10^9^	(17)
gallic acid	carboxyl, hydroxyl, aromatic	2.56 × 10^10^	(16)
succinic acid	carboxyl, alkane	3.1 × 10^8^	(17)
tryptophan	carboxyl, amines, aromatic	1.2–1.4 × 10^10^	(17)
trimesic acid	carboxyl, aromatic	4.3 × 10^8*^	*estimated here
coniferyl alcohol	methyoxy, aromatic, hydroxyl, alkene	6.98 × 10^9^	(18)

### Photocatalytic Degradation of *p*CBA

**Figure 2:**
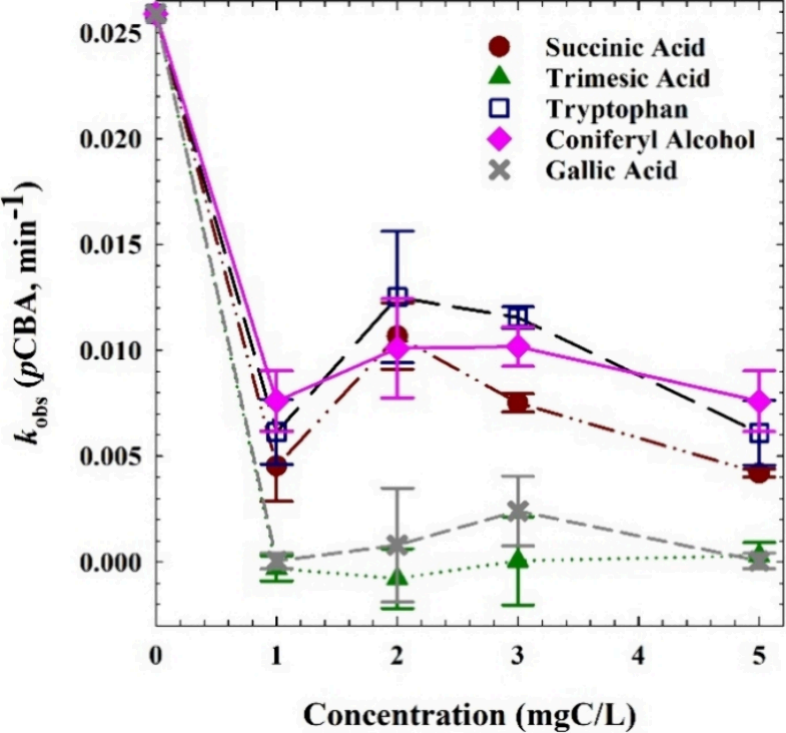
Photocatalytic *p*CBA degradation rate constants in the presence of 5 mg/L TiO_2_ and varying concentrations of inhibitory molecules.

The removal of *p*CBA was assumed to be first order with respect to an effective [OH·]_ss_, as demonstrated previously.^4,6^ The *k*_obs_ for each experimental condition are plotted as a function of inhibitor concentration in Figure 2, with kinetic profiles shown in Figure S3. This approach was instantiated by Brame et al. in 2015 as a method to discern the nature of competition for ROS in the photocatalytic system; a linear trend of *k*_obs_ versus scavenger concentration corresponds to bulk-phase reactions (as seen here in Figure 1) while a sharp, exponential decline in *k*_obs_ results from the role of Langmuir adsorption and competition for surface sites on the photocatalyst surface.^4^ This *k*_obs_ trend analysis provides a basis of comparison to check whether simulated interactions can predict photocatalytic inhibition. Our prior work showed that this approach was sensitive to changes in DOM composition from wastewater samples.^6,19^ Furthermore, the photocatalytic inhibition by DOM adsorbed onto TiO_2_ appeared to be determined by specific chemisorption dynamics, because inducing DOM-TiO_2_ aggregation by changing ionic strength did not predict inhibition outcomes while adjustments to pH caused substantial changes, especially across points of zero charge for TiO_2_ and *p*CBA.^7^ Notably, a clear uptick in photoactivity was observed for three of the compounds at about 2 mgC/L, followed by a decrease thereafter. This phenomenon has been documented elsewhere and is likely caused by attraction between the inhibiting molecules and *p*CBA, acting to draw the probe closer to the TiO_2_ surface as a secondary adsorption process.^5−7^ The inhibitory trends here show that each molecule competes with *p*CBA for surface phase reactions, with trimesic and gallic acids exerting the greatest inhibition followed by succinic acid, and then by coniferyl alcohol and tryptophan, which both performed similarly. The order of inhibition does not follow the pattern of reaction rate constants between each probe and OH·, indicating that reactions with OH· do not fully explain the inhibition. Rather, molecular affinities with the TiO_2_ surface likely dictate the inhibitory dynamics. The current literature on the reactivity of these molecules with electron holes (h^+^) is largely lacking. Although tryptophans are known to be an effective h^+^ transporter in biological systems and may be less susceptible to degradation by h^+^,^20^ competition for active surface sites still controls *p*CBA exposure to h^+^. The importance of the surface-phase interactions should not be underestimated; indeed, these dynamics are the reason multiactivity assessments have become widespread for the evaluation of photocatalytic performance.^8^

The challenges to accurately compare photocatalytic performances across different materials—such as variations in laboratory capabilities and photochemical testing procedures—necessitate the development of strategies to characterize photoactivity from a fundamental or first-principles perspective. To this end, we selected inhibitory molecules with diverse molecular characteristics (aromatic, carboxy, hydroxy, and amine groups) to systematically investigate their specific interactions with TiO_2_ and their effects on the photocatalytic degradation of *p*CBA. However, their moiety composition alone cannot fully account for the observed inhibitory action across these molecules. Notably, molecules such as trimesic and gallic acid, each with three substituents around a benzene ring, exhibit a radial symmetry of electronegative groups, which provides a tentative rationale for their distinct competitive behavior. This observation underlines the complexity of molecular interactions on photocatalytic surfaces, requiring further investigation to validate such hypotheses and elucidate the nuanced effects of molecular structure on adsorption. Furthermore, it is important to note that the selection of inhibitory molecules here is representative but not all-inclusive. Additional studies with a broader range of organic compounds are necessary to comprehensively understand the diverse interactions that can influence photocatalytic performance.

### Surface Interactions

Each of the molecules investigated was simulated with an anatase TiO_2_(101) surface and solvated in a water system using an implicit solvation model. The TiO_2_ surface was modeled as a 10.87 × 11.33 Å periodic slab with a 20 Å separation between slabs. The surface contained six exposed Ti atoms available for interaction. Surface interaction energies were calculated in gas (*E*_int_^gas^) and aqueous (*E*_int_^aq^) phases, as reported in Table 2. Gas phase simulations show that *p*CBA has an *E*_int_^gas^ of −2.28 eV with the TiO_2_ surface; trimesic acid had a significantly stronger affinity at −3.82 eV, followed by gallic acid at −2.81 eV. Succinic acid and tryptophan also exhibited slightly stronger attraction to the surface than *p*CBA in the gas phase, and coniferyl alcohol had the weakest interactions at −1.66 eV. The comparative energies match the observed inhibitory profiles (Figure 2) well, given that trimesic and gallic acids were most competitive with *p*CBA for active surface sites. Aqueous phase interaction energies showed a similar condition except that gallic and succinic acids were the only molecules with stronger affinities for the TiO_2_ surface in solution, while *E*_int_^aq^ for trimesic acid (−1.10 eV) was near that of *p*CBA (−1.14 eV). The weak interaction energies for tryptophan and coniferyl alcohol match the observed competition since these exerted the weakest inhibition. Conversely, *E*_int_^aq^ of succinic acid suggested a stronger interaction with the surface than trimesic acid. Nevertheless, the experimental results showed a weaker inhibitory effect (Figure 2). This discrepancy may be due to the comparatively poor reactivity with OH·.

**Table 2:** Surface Interaction Energies for the Probe and Competitor Molecules with a Simulated TiO_2_(101) Surface

molecule	*E*_int_^gas^ (eV)	*E*_int_^aq^ (eV)
tryptophan	–2.31	–0.54
coniferyl alcohol	–1.66	–0.67
succinic acid	–2.56	–1.28
gallic acid	–2.81	–1.30
trimesic acid	–3.82	–1.10
*p*CBA	–2.28	–1.14

**Figure 3:**
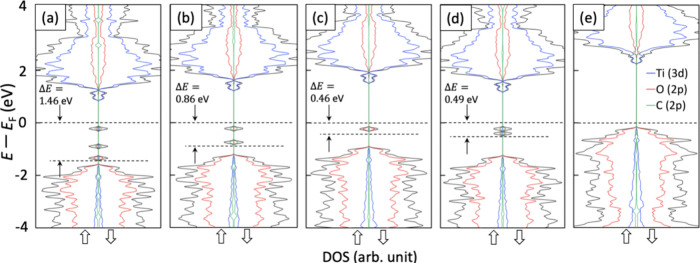
Electronic density of states (DOS) plots for the Ti(3d), O(2p), and C(2p) orbitals for TiO_2_ with (a) coniferyl alcohol, (b) tryptophan, (c) succinic acid, (d) gallic acid, and (e) trimesic acid.

The density of states (DOS) of TiO_2_ and adsorbate molecules affords a deeper assessment of the character of the surface interactions. We calculate a band gap, *E*_g_, of 2.42 eV, which is commonly reported in the theoretical literature for anatase.^21^ However, we suspect that the calculated trends offer meaningful insights. As shown in Figure S4, adding *p*CBA to the surface (Figure S4b) did not induce changes in the electronic states of TiO_2_, which is contrasted with the inhibitory molecules (except for trimesic acid) resulting in the formation of distinct electronic states, as shown in Figure 3. The distribution of electronic states for trimesic acid (Figure 3e) was similar to that of *p*CBA with a high density near the Fermi level, which is in line with the experimental observation that trimesic acid exerted the strongest competition for surface sites. The newly formed states on the remaining molecules (Figure 3a–d) occurred below the Fermi level and had low population densities relative to the valence states. The spatial distributions of these states are illustrated in Figure S5. Although *E*_g_ is negligibly changed, the new states widen the gap, Δ*E*, between the Fermi level and the VBM, as illustrated in Figure 3. Except for gallic acid, we see a consistent trend between the width of Δ*E* and inhibitory trends. Coniferyl alcohol (Figure 3a) yields three distinct states upon adsorption to TiO_2_, forming the largest Δ*E* of the set, followed by tryptophan with two distinct states (Figure 3b). The new interaction states for succinic and gallic acids (Figure 3c,d) were closer to the Fermi level, and gallic acid had two states that were tightly grouped, affording more density near the Fermi level than coniferyl alcohol, tryptophan, or succinic acid. Assuming that electron density near the Fermi level is an important predictor of adsorptivity,^22^ these DOS observations indicate an approximate order of adsorption affinity: *p*CBA ≈ trimesic acid > gallic acid > succinic acid > tryptophan > coniferyl alcohol. This analysis agrees exceptionally well with the experimental observations, where trimesic and gallic acids were similarly competitive with *p*CBA, followed by succinic and then the remaining two (Figure 2). Interestingly, both the inhibitory trends, as well as the width of Δ*E*, generally align well with the calculated *E*_int_^gas^, which delineates the strength of the interaction between the adsorbate and the surface by factoring out the energies associated with surface and molecular reconstruction.

In addition to our primary focus on the anatase TiO_2_(101) surface, which is expected to compose 94% of the anatase surface,^23^ we have performed additional calculations for the (001) surface. Although less prevalent, the (001) surface is known to be significantly more reactive.^23,24^ We focused on the probe molecule as well as one of the more strongly inhibiting and weakly inhibiting molecules: trimesic acid and tryptophan, respectively. As seen by the adsorption energies, *E*_ads_, in Table S1, trimesic acid and the probe in particular exhibit much stronger adsorption on the (001) than on the (101) surface. Specifically, trimesic acid shows a *E*_ads_ of −3.84 eV on the (001) facet compared to approximately −2.40 eV on the (101) facet. Similarly, the probe molecule exhibits an *E*_ads_ of −2.38 eV on the (001) surface versus −1.57 eV on the (101) surface. Further, we find a similar trend on the (001) surface where trimesic acid is much more strongly adsorbed than tryptophan and the probe. This suggests a similar inhibition of probe access to the (001) surface as observed on the (101) surface but with a more pronounced effect due to the higher reactivity of the (001) facet. Furthermore, similar to trends observed in Figure 3, the presence of tryptophan shifts the Fermi level away from the VBM for the (001) facet, as seen in Figure S6. On the other hand, trimesic acid, whose electronic states are more hybridized with those of TiO_2_, introduces more significant changes near the Fermi level.

### Machine-Learning Accelerated Explicit Solvation Model

Although the overall experimentally observed inhibitory trends aligned quite closely with *E*_int_^gas^, the *E*_int_^aq^, calculated using an implicit solvation model, deviated from expectations, particularly in the cases of trimesic acid and *p*CBA. As seen in Table 2, *E*_int_^gas^ values suggest that trimesic acid should have a stronger interaction with the surface than the probe, which would explain its substantial inhibition of *p*CBA degradation. Nevertheless, the overall trend captured by the implicit solvation model indicated that tryptophan and coniferyl acid can be categorized in a class of their own in terms of the interaction strength with the surface relative to the other species, with the other molecules having relatively similar *E*_int_^aq^ values. Well-documented shortcomings of implicit solvation motivated the use of an explicit model of water molecules to better understand the system. As shown by Camellone and co-workers, solvation effects on the anatase TiO_2_ surface can be accurately and thoroughly described utilizing ab initio molecular dynamics (AIMD).^21^ Such explicit solvation models necessitate substantially greater computational resources than the implicit models and often require 20 ps simulation times (or longer depending on complexity) to attain relatively equilibrated energetics.

**Figure 4:**
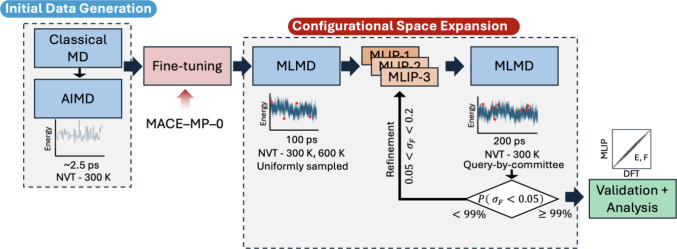
Illustration of the MLIP training workflow.

Here, we turn to a similar explicit solvation model. To address the substantial computational cost of the ab initio simulations required to model the explicit solvation environment and to capture statistically meaningful trends over extended time scales, we employed machine learning interatomic potentials (MLIPs) through equivariant graph neural networks, utilizing the MACE architecture.^25^ These MLIPS have been shown to achieve high accuracy with respect to quantum mechanical calculations, particularly when provided with a sufficiently diverse and relevant data set.^26^ Therefore, we assembled our explicit solvation models and performed preliminary equilibration utilizing classical molecular dynamics (MD), followed by AIMD simulation to generate an initial data set (see Figure 4). The initial data set consisted of ∼15,300 configurations, which included trimesic acid, tryptophan, and *p*CBA over the (101) anatase surface in the aqueous solvation condition, in addition to condensed state molecular configurations comprising combinations of water molecules with the three molecules to increase the data diversity. This data set was used to finetune the MACE-MP-0^27^*foundation model*, which was originally pretrained on >1.5 million configurations from the Materials Project.^28^ The finetuned MLIPs, which accelerate the simulations by several orders of magnitude, allowed us to explore configurations over time scales greater than 100 ps—far exceeding the ∼2.5 ps limit of our AIMD simulations. We initially selected up to 200 configurations over a 100 ps simulation for each of the three adsorbate molecules. In subsequent refinement stages, an active learning pipeline using a query-by-committee approach was instituted to ensure the robustness of our simulations. This approach ensured that additional configurations were strategically selected for labeling, as necessary, if an ensemble of MLIPs demonstrated high variance in force prediction of new geometries.

**Figure 5:**
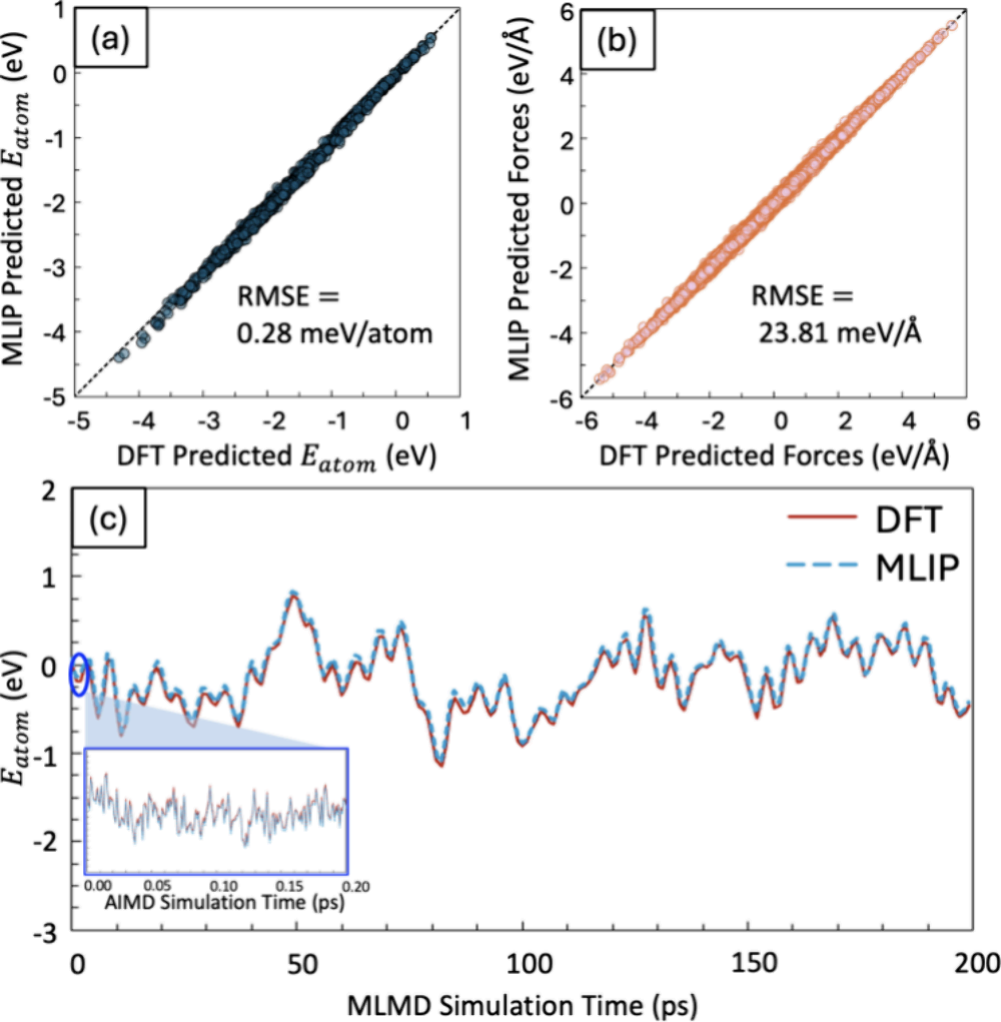
Parity plots illustrating MLIP predictions versus DFT calculations for (a) atomization energies and (b) forces across a set of geometries generated during MLMD simulations, which extend beyond the test data from the initial splits. (c) DFT labels on MLMD generated geometries over the course of a 200 ps simulation, by labeling 200 frames using DFT. The inset depicts MLIP labels on AIMD generated geometries over the last 200 fs of a 2.7 ps simulation.

Models trained through the MLIP workflow, as seen in Figure 5a,b, achieved a mean absolute error (MAE) of 75 meV (<2 kcal/mol) and 16.1 meV/Å or better for energy and forces, respectively, on the unseen data generated by the machine learning molecular dynamics (MLMD) simulations. This performance indicated that the models predict energy and forces within chemical accuracy. In the final stage (200 configurations), each adsorbate–slab complex was uniformly sampled and labeled from the production stage simulations as an additional layer of validation for the MLIP accuracy on the new configurations, as shown in Figure 5c.

**Figure 6:**
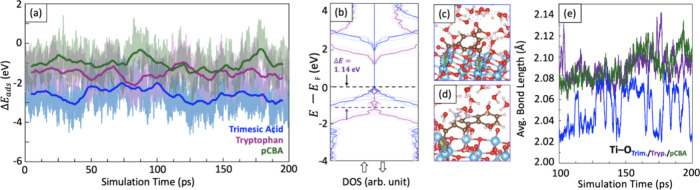
(a) Relative adsorption energy trends over the course of a 200 ps MLMD simulation. (b) Averaged DOS from sampled geometries for (c) trimesic acid– and (d) tryptophan–TiO_2_ complexes. (e) Averaged bond lengths for the three adsorbates with the Ti atoms on the anatase surface.

To evaluate the adsorption behavior of tryptophan, trimesic acid, and *p*CBA on the anatase surface, relative adsorption energies, Δ*E*_ads_, were evaluated by the following equation:
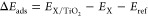
where *E*_X/TiO_2__, *E*_X_, and *E*_ref._ Are the energies of the surface with the adsorbate in the explicit solvation, the adsorbate in the gas phase, and an arbitrary reference energy (here *E*_ref_ is set as the time-averaged energy for a reference TiO_2_ surface with 62 water molecules). As depicted in Figure 6a, time-averaged Δ*E*_ads_ for trimesic acid and tryptophan on the TiO_2_ surface are 1.58 and 0.51 eV lower, respectively, than that of the probe molecule. This observation suggests that trimesic acid, in contrast to tryptophan, forms a substantially stronger interaction with the TiO_2_ surface, resulting in the nearly complete inhibition of probe molecule degradation at higher trimesic acid concentrations. Furthermore, in agreement with prior work, our simulations show that water molecules saturate the coordinatively unsaturated sites of Ti atoms on the anatase surface, as seen in Figure 6c,d. Therefore, the inhibitory molecules and the probe do not merely compete with each other; rather, both compete with water for surface adsorption. Trimesic acid exhibited a distinct adsorption mechanism on the anatase surface due to its tricarboxylic acid functionality. Its three carboxyl groups typically deprotonate, and subsequently, two of the groups coordinate with surface Ti atoms. This bidentate binding mode significantly enhances the stability of the adsorption complex formed with the anatase surface. This multivalent interaction contrasts sharply with the adsorption behavior of tryptophan or the probe molecule, each of which possesses a singular carbonyl group that facilitates monodentate attachment to the surface. Consequently, trimesic acid is more likely to block available adsorption sites, preventing the degradation target from approaching the surface and accessing generated OH· radicals near the surface.

The competitiveness of trimesic acid adsorption was further evidenced by averaged DOS calculations (Figure 6b) derived from sampled configurations over the last 50 ps of the simulations. This analysis exhibited patterns consistent with those observed in the gas phase calculations. Specifically, electronic states associated with trimesic acid were completely hybridized with the anatase valence states near the Fermi level, indicative of a strong chemisorptive character. In contrast, tryptophan displayed a distinct behavior with the formation of midgap states that modulated the anatase valence states by a shift in energy (Δ*E*) relative to the Fermi level. Furthermore, the longer Ti–O adsorbate bond lengths observed for tryptophan corroborated the distinctive binding behavior, indicating looser interactions with the surface (see Figure 6e). These observations align with tryptophan’s reduced, rather than prohibitive, impact on photocatalytic degradation.

### Charge Redistribution

**Figure 7:**
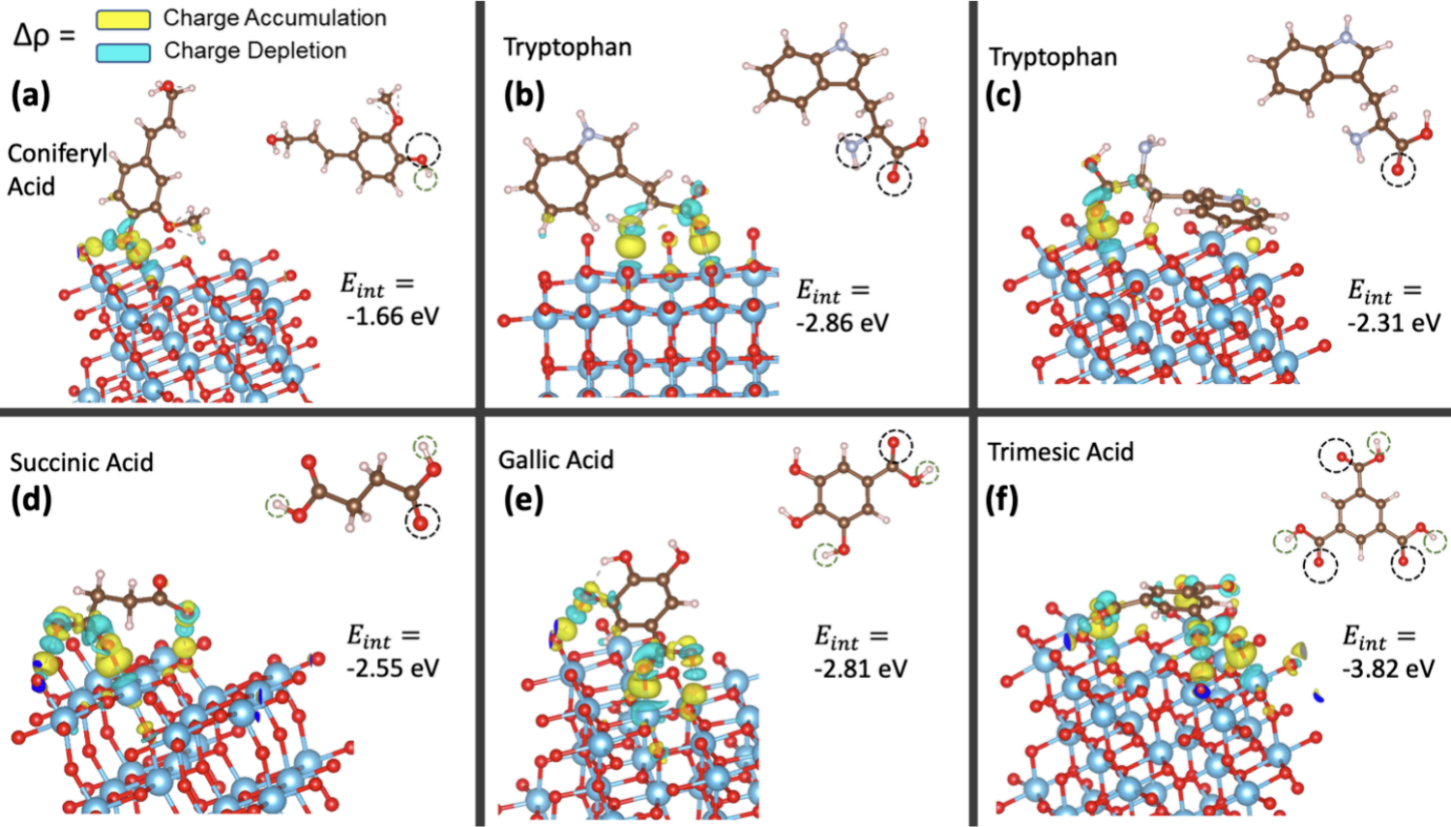
Depiction of partial charge accumulation (yellow) and depletion (cyan) regions for (a) coniferyl alcohol, (b) tryptophan in a vertical orientation with respect to TiO_2_, (c) tryptophan in a flat orientation with respect to TiO_2_, (d) succinic acid, (e) gallic acid, and (f) trimesic acid.

In complement to the DOS analysis, partial charge redistributions were calculated for the TiO_2_–adsorbate systems, as depicted in Figure 7. In general, each molecule underwent charge depletion at an oxygen atom and at least one hydrogen atom, affording a corresponding charge accumulation at the electronegative oxygens of either TiO_2_ or the adsorbate molecule. A corresponding charge depletion occurred at the Ti and H atoms interacting with O. An exception to the general charge transfer pattern is observed with tryptophan. In its vertical configuration, tryptophan displays partial charge depletion at its nitrogen atom instead of at its hydrogen atom (Figure 7b). Conversely, in the flat configuration, which is more likely based on its lower adsorption energy (*E*_ads_) reported in Table S1, no charge transfer occurs with any hydrogen atom (Figure 7c). Tryptophan and coniferyl alcohol interacted with TiO_2_ at only one edge of their respective molecular structures. The other three molecules experienced charge transfer interactions at opposing ends of the molecule. Trimesic acid, in particular, exhibited charge accumulation at each of its three carbonyl oxygens, indicating significant interactions. These patterns of charge transfer are consistent with the interaction energies reported in Table 2: tryptophan and coniferyl alcohol exhibited the least favorable interaction energies. The broader surface coverage by molecules with more favorable interaction energies suggests a competitive mechanism for surface adsorption sites. Considering that succinic acid showed minimal reactivity with OH· in the bulk phase (Table 1 and Figure 1), its strong competitive effect versus *p*CBA observed in Figure 2 is likely due predominantly to its effective adhesion to the TiO_2_ surface. Similarly, trimesic acid transitions from being a weaker inhibitor in bulk solution to being the most inhibitory on TiO_2_ surfaces due to its effective surface interactions.

## Conclusions

Despite the progress in various aspects of photocatalytic materials, one opportunity for optimization has been largely overlooked for their application to water treatment. Little attention has been given to tuning surface characteristics for improved substrate-specific interactions to maximize photocatalytic activity, especially in the face of interfering cosolutes. Most efforts to characterize differential surface activity in photocatalytic systems have been focused on establishing appropriate bases of comparison. In 2008, Ryu and Choi asserted the importance of using multiactivity tests when comparing photocatalytic materials because differences in doping, surface morphology, particle size, and other characteristics influence photocatalytic performance. However, this challenge is also a potential design feature. The experiments presented here offer an alternative multiactivity assessment protocol to screen photocatalyst–solute pairings for competitive activity.

DFT calculations demonstrate a strong correlation between the interaction energies of anatase surfaces with various inhibitory molecules and the experimental observations in the probe–quencher competition. This analysis indicates that adsorption site interactions overshadow the role of the general reactivity with OH· radicals. Furthermore, ML-accelerated explicit aqueous solvation simulations reveal that water molecules saturate the anatase active sites, indicating that inhibitory cosolvents and the probe not only compete with each other but also with water for adsorption on the TiO_2_ surface. Further, molecules with multiple functional groups, such as trimesic acid, exhibit enhanced adhesion to TiO_2_ surfaces, resulting in substantial inhibition of photocatalytic activity. For instance, trimesic acid, with its tricarboxylic acid functionality, demonstrates a distinct adsorption mechanism by deprotonating and coordinating two of its carboxyl groups with Ti atoms on the surface. This bidentate binding mode significantly enhances the stability of the adsorption complex.

In summary, this study demonstrates that the integration of DFT and MLIPs offers a robust framework for predicting and optimizing surface interactions in photocatalytic materials. This approach may serve as a useful framework for predicting and optimizing photocatalyst performance for more effective environmental remediation technologies.

## Methods/Experimental Section

### Materials and Chemicals

All chemicals used were analytical or HPLC grade, used without further purification. Ultrapure water (>18.0 MΩ·cm) was used for the preparation of reagent and experimental solutions. Anatase TiO_2_ nanoparticles were procured from Alfa Aesar (Tewksbury, MA) with a nominal particle diameter of 32 nm and a bulk surface area of 45 m^2^/g. Hydroxyl radicals were quantified by using *para*-chlorobenzoic acid (*p*CBA; Alfa Aesar) as a probe molecule. Succinic acid, gallic acid, coniferyl alcohol, tryptophan, and trimesic acid were all obtained from Sigma-Aldrich (Burlington, MA).

### Photochemical Experiments

Photochemical reactions were conducted in a ventilated photoreactor cabinet with a magnetically stirred reactor vessel at room temperature. An LG Innotek 6060 (LG Innotek Co., Ltd., Seoul, South Korea) UV_278_ LED lamp was used as an excitation source for the TiO_2_ and for photolysis of H_2_O_2_ for bulk phase OH· production, with a distance of 20 cm between the lamp and the experimental solution. The irradiance at the surface of the reactor solution was measured to be 278 μW/cm^2^ with a UVX radiometer (UV-25 attachment, Analytik Jena, GmbH). The photochemical activity was monitored using *p*CBA as a probe molecule, sensitive to the reaction with OH·. In all experiments, 10 μM *p*CBA was the initial condition, with its concentration monitored at 234 nm over time by using an HPLC-UV system (Agilent Technologies, Inc., 1260 Infinity). A reverse phase C18 column was used with a 40:60 mixture of acetonitrile to phosphoric acid solution, as reported previously.^7^ Competitor molecules were added, individually, to experiments at concentrations ranging from 1 to 5 mg/L to observe moiety-specific competition for OH· and active TiO_2_ surface sites.

### Computational Methods

DFT calculations were performed using the Vienna Ab initio Simulations Package (VASP)^29^ with the projector augmented wave (PAW) pseudopotential with a cutoff of 400 eV. The exchange–correlation interaction was described using the generalized gradient approximation (GGA) Perdew–Burke–Ernzerhof (PBE) functional with the projector augmented wave (PAW) method.^30^ Strong on-site Coulomb interactions were accounted for using Hubbard parameter *U* = 4.2 eV for the Ti(3d) orbital. We primarily focus our analysis on the TiO_2_(101) anatase surface, which was modeled as a 10.87 Å × 11.33 Å periodic slab (3 × 4 TiO_2_ units) with 20 Å separation between slabs, exposing six Ti atoms on the surface available for binding as shown in Figure S7. The slabs comprised three layers, with the bottom layers fixed to model bulk TiO_2_. A 4 × 4 × 1 *k*-point Monkhorst–Pack mesh was used to simulate the anatase surface. The van der Waals interactions were described by implementing DFT-D3 with Becke–Johnson damping.^31^

The adsorbate molecules were introduced to the TiO_2_(101) surface in two primary configurations. Namely, vertical and flat starting orientations of adsorbates with the surface were optimized to find the most favorable configurations, as shown in Figure S8. TiO_2_–adsorbate interaction energies, *E*_int_, were computed according to
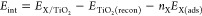
where *E*_X/TiO_2__ is the energy of the anatase surface with *n* adsorbed molecules, *E*_TiO_2_(recon)_ is the energy of the bare (reconstructed) anatase surface, and *E*_X(ads)_ is the energy of the isolated molecule, X*,* in its adsorbed geometry in the simulation box. In this study, we evaluate *E*_int_ both in the gas phase and in the presence of the implicit (aqueous) solvent. Furthermore, the adsorption energy, *E*_ads_, was also defined as
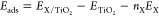
where *E*_TiO_2__ and *E*_X_ are the energies for the relaxed bare surface and relaxed isolated molecule in the gas phase. Correspondingly, *E*_ads_ can be connected to the *E*_int_, which specifically relates to the energy contribution toward bond formation, by considering the energy necessary for the reconstruction of the surface and the molecule, X, upon their interaction. Here, *E*_ads_ (reported in Table S1) is used to assess the overall thermodynamic favorability of each molecule’s relaxed configuration on the surface. Interestingly, we found that *E*_int_ provided more direct insight into the strength of the adsorption process and interaction, corresponding rather strongly with the experimentally observed inhibitory trends, as well as the electronic density of states.

To provide a more granular analysis of these interactions, incorporating an energy decomposition analysis (EDA) would be advantageous. EDA would enable the separation of the total interaction energy into its fundamental components, such as electrostatic, exchange–correlation, polarization, and dispersion interactions, offering a deeper understanding of the forces driving adsorption and surface chemistry. Such analysis can be incorporated in future work to shed further light on the adsorbate–surface interactions and inhibitory trends observed.

For our MLIPs, we utilize the MACE architecture^25^ with the small MACE-MP-0^27^ pretrained *foundation model* as a starting point, as it has shown excellent performance on a wide range of materials and applications, well outside of its underlying training set of the MPtrj data set. The model consists of a 128-size vector for atomic features with interactions described through four-body correlations within each layer. A radial cutoff of 6 Å was used. Model training was executed using the Adam optimizer with an initial learning rate of 0.01. The batch size for training was set at 8, using an exponential moving average with a decay factor of 0.99. The initial data set was divided into a 90:5:5 ratio for training, validation, and testing, respectively. Weights for energy and force losses were initially set to 1 and 10, respectively.

The explicit solvation model incorporated 62 water molecules, which correspond to the approximate number of water molecules needed to achieve a density of 1 g/cm^3^ in the gap between TiO_2_ slabs. *NVT* simulations at 300 K were performed, using the Universal force field, to obtain a reasonable starting point. This step was followed up by ∼2.5 ps AIMD *NVT* simulations, performed at 300 K, using the Γ-point for electronic integrations. Following this, all MLMD simulations were performed using the Langevin *NVT* framework at 300 K.

An enlarged model with an expanded TiO_2_ surface (6 × 8 TiO_2_ units) and two adsorbates was also simulated, with over 1200 atoms, to investigate the effect of halving the concentration. As seen in Figure S9, this model yielded less accurate energy predictions, though critically, the trends were maintained (due to a consistent underestimation of energies) despite the models not being explicitly trained on these systems, which inherently introduces an expanded configurational space beyond what the model has seen. Similar to the analysis shown in Figure 6a, trimesic acid consistently showed a much lower Δ*E*_ads_ (more substantial interaction with the surface) than both the probe and tryptophan, which showed similar Δ*E*_ads_.

## Supporting Information Available

Molecular diagrams, photocatalytic *p*CBA degradation kinetic profiles, visualizations of computed spatial distributions of charge states of TiO_2_ with adsorbates, depictions of adsorbate conformations with TiO_2_ surface, depictions with dimensions of simulated TiO_2_ slab, and a tabulation of calculated adsorption energies between TiO_2_ and adsorbed molecules (PDF)
