# Supplementary material for: Unveiling Competitive Adsorption in TiO2 Photocatalysis through Machine-Learning-Accelerated Molecular Dynamics, DFT, and Experimental Methods
Source: ACS Appl Mater Interfaces. 2024 Jul 2;16(28):36215–23. doi: 10.1021/acsami.4c02334 (PMC11261558; doi:10.1021/acsami.4c02334)
Supplement: Supplementary file 1 — am4c02334_si_001.pdf [file am4c02334_si_001.pdf]

Supporting Information

Title: Unveiling Competitive Adsorption in TiO_2 Photocatalysis through Machine Learning-Accelerated Molecular Dynamics, DFT, and Experimental Methods

Authors: Omar Allam[1,2†], Mostafa Maghsoodi[3†], Seung Soon Jang[2*], Samuel D. Snow[3*]

[1]Woodruff School of Mechanical Engineering, Georgia Institute of Technology, Atlanta, GA 30332, USA

[2]Computational NanoBio Technology Laboratory, School of Materials Science and Engineering, Georgia Institute of Technology, Atlanta, Georgia, USA

[3]Department of Civil and Environmental Engineering, Louisiana State University, 3255 Patrick Taylor Hall, Baton Rouge, Louisiana 70803, USA

*Corresponding authors: Dr. Seung Soon Jang (seungsoon.jang@mse.gatech.edu) and Dr. Samuel Snow (ssnow@lsu.edu);

[†]These authors contributed equally

Figures: 9
Tables: 1
Pages: 8

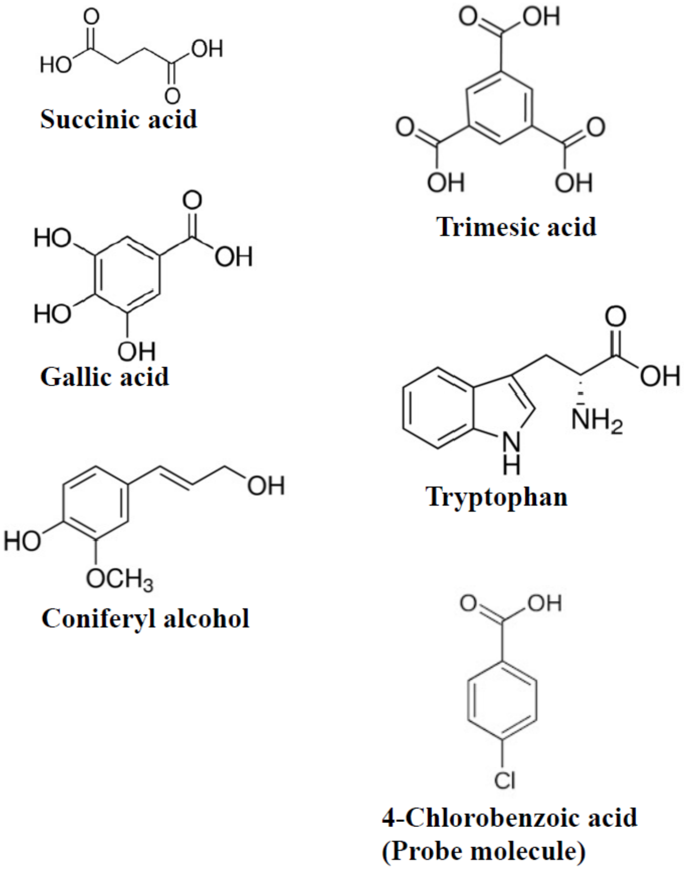

Figure S1. Molecular diagrams for probe and competitor molecules.

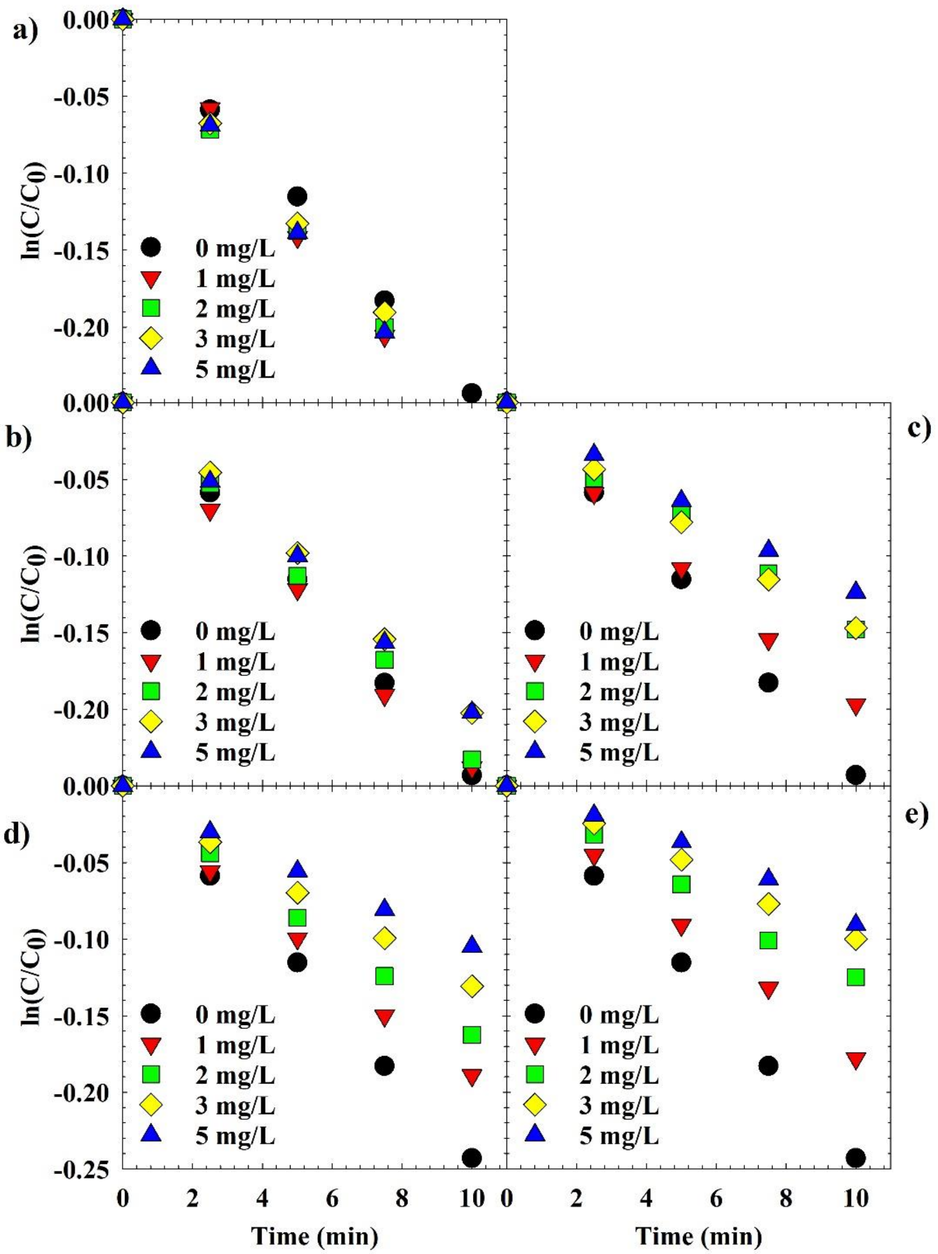

Figure S2. Kinetic profiles for *p*CBA destruction by OH• produced by H_2O_2 (1%) photolysis under 278 nm irradiation in the presence of varying concentrations of a) succinic acid, b) trimesic acid, c) tryptophan, d), coniferyl alcohol, or e) gallic acid.

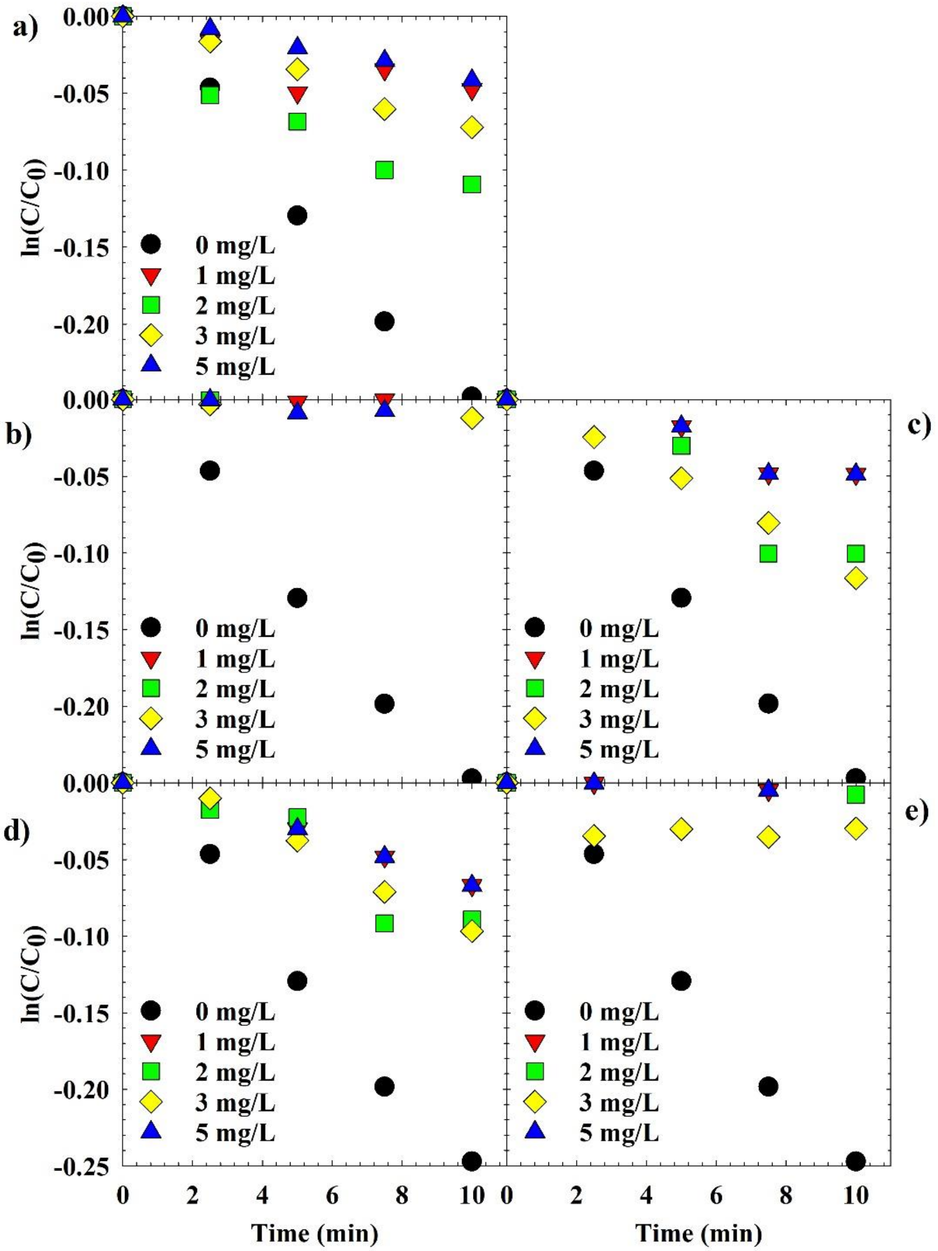

Figure S3. Kinetic profiles for *p*CBA destruction by TiO_2 (5 mg/L) photocatalysis under 278 nm irradiation in the presence of varying concentrations of a) succinic acid, b) trimesic acid, c) tryptophan, d), coniferyl alcohol, or e) gallic acid.

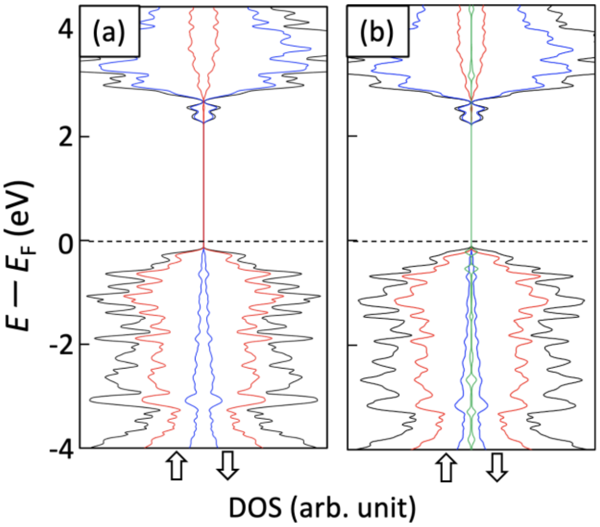

Figure S4. Electronic density of states plots for the Ti(3d), O(2p) and C(2p) orbitals for **a**) TiO_2 with no adsorbate and **b**) TiO_2 with *p*CBA. The dashed line across the zero position of the y axis marks the Fermi level.

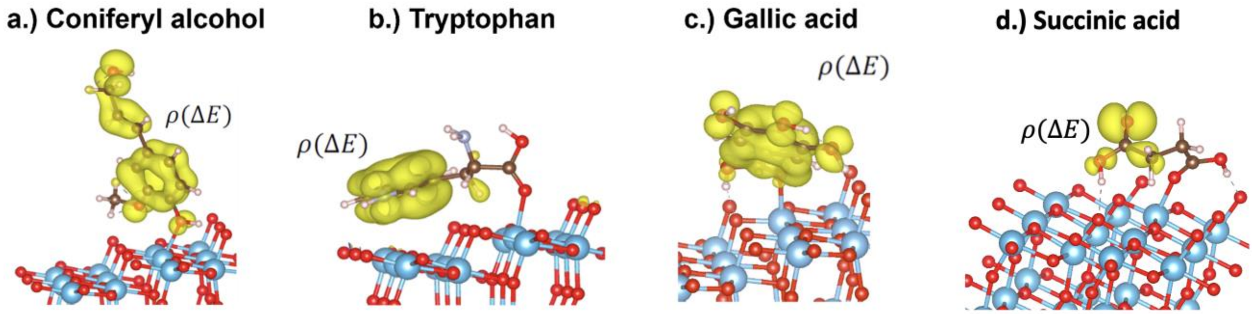

Figure S5. Spatial distributions of charge density in the newly developed states, ΔE, of **a**) coniferyl alcohol, **b**) tryptophan, and **c**) gallic acid, and **d)** succinic acid.

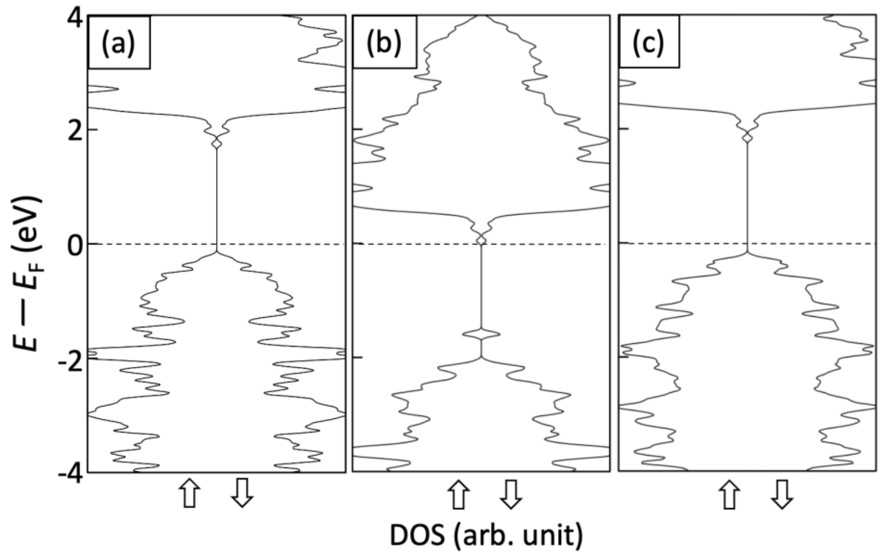

Figure S6. Electronic density of states for **a**) the bare (001) anatase surface, **b**) the (001) anatase surface with tryptophan, and **c**) the (001) anatase surface with trimesic acid.

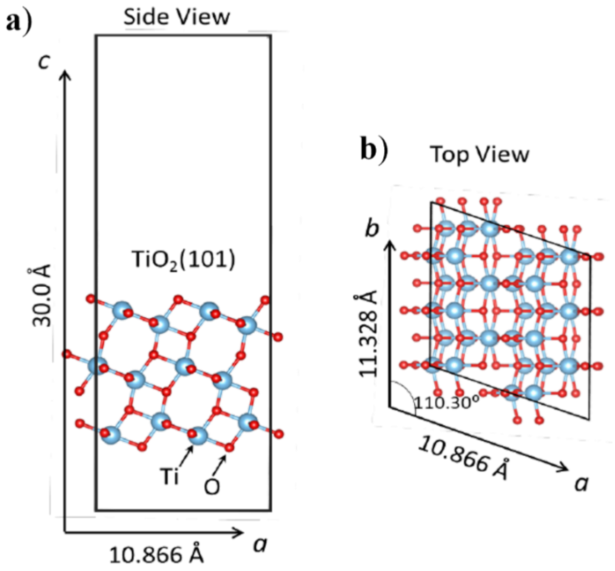

Figure S7. Bulk anatase TiO_2 slab system shown with **a**) a side view with vacuum space and **b**) a top-down view of the TiO_2 surface.

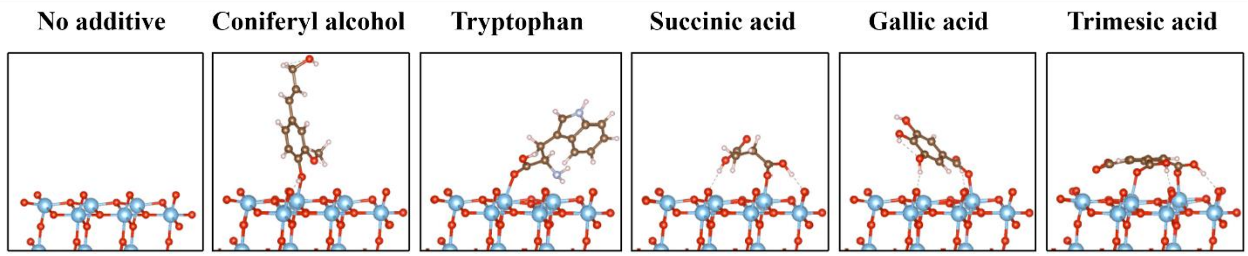

Figure S8. TiO_2 surface with or without adsorbed competitor molecules.

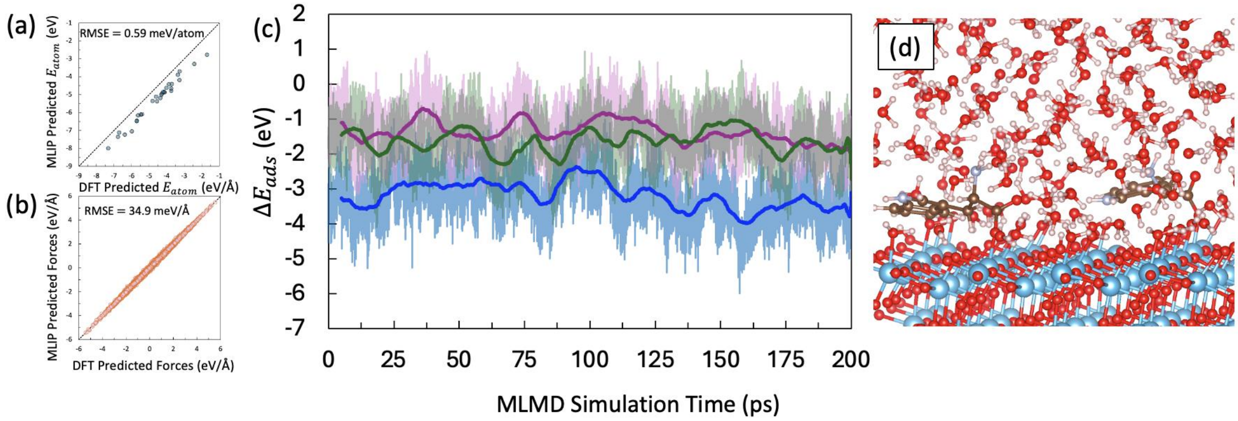

Figure S9. Parity plots for **a**) energy and **b**) force predictions of the adsorbates over the 6x8 surface. (**c**) Adsorption energy evolution (per adsorbed molecule) over the course of the simulation. The adsorption energies for the probe, tryptophan, and trimesic acid are depicted by green, purple, and blue, respectively. (**d**) Illustration of the adsorbate-slab explicit solvation model.

Table S1. DFT and calculated binding energies for adsorbate molecules in interacting with anatase TiO_2 with flat and vertical configurations. All cases correspond to the (101) surface unless otherwise specified. *Adsorbate Coverage* refers to the number of adsorbate molecules per 3×4 TiO_2 surface.

Molecule	Initial Configuration	Adsorbate Coverage	E_{ads} (eV)
Succinic acid	Vertical	1	-1.88
Succinic acid	Flat	1	-1.78
Succinic acid	Flat	2	-1.79
Trimesic acid	Vertical	1	-2.41
Trimesic acid	Flat	1	-2.35
Trimesic acid (001)	Flat	1	-3.84
Tryptophan	Vertical	1	-2.04
Tryptophan	Flat	1	-2.43
Tryptophan	Vertical	2	-2.45
Tryptophan (001)	Flat	1	-2.35
Coniferyl alcohol	Vertical	1	-1.30
Coniferyl alcohol	Vertical	2	-1.52
Gallic acid	Vertical	1	-1.91
Gallic acid	Flat	1	-1.74
Gallic acid	Vertical	2	-1.89
Gallic acid	Vertical	3	-1.79
Probe	Vertical (Cl – TiO_2)	1	-0.44
Probe	Vertical (COOH – TiO_2)	1	-1.21
Probe	Flat	1	-1.57
Probe (001)	Flat	1	-2.38
